# Improved Post‐Processing Framework for Whole Slide Imaging and 3D Reconstruction

**DOI:** 10.1002/jemt.70164

**Published:** 2026-07-21

**Authors:** Emily L. De Leon Alvarado, Daryan Chitsaz, Ilana R. Leppert, Jennifer S. W. Campbell, Yangyingqi Zhou, G. Bruce Pike, Timothy E. Kennedy, Christine L. Tardif, Christopher D. Rowley

**Affiliations:** ^1^ Department of Physics and Astronomy McMaster University Hamilton Canada; ^2^ Department of Neurology and Neurosurgery McGill University Montreal Canada; ^3^ McConnell Brain Imaging Centre Montreal Canada; ^4^ Department of Psychology, Neuroscience & Behavior McMaster University Hamilton Canada; ^5^ Hotchkiss Brain Institute University of Calgary Calgary Canada; ^6^ Department of Radiology University of Calgary Calgary Canada; ^7^ Department of Clinical Neuroscience University of Calgary Calgary Canada; ^8^ Department of Biomedical Engineering McGill University Montreal Canada

**Keywords:** image reconstruction, level‐set method, tile stitching, vignette correction, whole slide imaging

## Abstract

Whole slide imaging (WSI) enables high‐resolution digitization of histological sections, but 3D reconstruction from serial slides remains hindered by illumination artifacts, tile inconsistencies, and section‐specific geometric distortions. We present an enhanced reconstruction framework that addresses both tile‐level intensity inhomogeneity and instability in deformable registration. First, we introduce an overlap‐aware illumination correction that refines an initial flat‐field estimate by adding an intensity equality constraint in overlapping tile regions. Second, intensity normalization of sections was performed before reconstruction to ensure consistent intensity profiles among serial sections. Lastly, we incorporate level‐set method (LSM) constraints into an existing volumetric reconstruction framework to stabilize alignment. The framework was developed for marmoset brain histology, including within‐specimen blockface photography and magnetic resonance imaging (MRI). The LSM reconstruction was validated using a synthetic banana image with a known initial shape and with mouse histology reconstructed using an external 3D MRI reference. Overlap‐aware correction in the marmoset WSI significantly reduced residual gridding artifacts across 112 sections (Friedman *χ* ^2^(2) = 890.04, *p* < 2.2 × 10^−16^). Within the marmoset dataset, intensity normalization reduced inter‐slice intensity variability (coefficient of variation: 0.392 to 0.246) and incorporation of LSM consistently improved registration accuracy by approximately 10% in Normalized Mutual Information scores and 3%–5% in Structural Similarity Index Measure. LSM also yielded improved structural correspondence in both gray matter and white matter structures.

## Research Highlights

- Developed a reproducible WSI post‐processing pipeline for accurate 3D histological reconstruction.
- Implemented overlap‐aware illumination correction to reduce residual gridding artifacts.
- Applied intensity normalization to improve inter‐slice consistency in serial sections.
- Incorporated level‐set method constraints to enhance registration accuracy and structural correspondence.

## Graphical abstract

Workflow for 3D reconstruction of marmoset whole‐slide histology. Individual tiles undergo vignette correction to address illumination artifacts before stitching into full‐section images. Sections are then reconstructed using the Possum framework, with a level‐set method incorporated at the nonlinear deformable stage to stabilize alignment and reduce common histological artifacts.

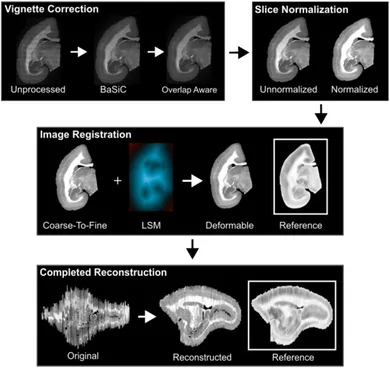

## 1 Introduction

Whole slide imaging (WSI) has become a crucial modality in modern histopathology, enabling the digital conversion of tissue on glass slides into high‐resolution virtual slides with magnifications ranging from 2× to 100×, for the entire tissue section (Higgins 2015; Kumar et al. 2020). Beyond facilitating precise, large‐scale visualization, WSI supports the reconstruction of three‐dimensional (3D) volumes from serial histological sections, providing new opportunities to study the microstructural organization of complex tissues such as the brain, liver, lung, cervix, and breast (Bongaerts et al. 2018; Deutsch et al. 2025; Ghaznavi et al. 2013; Melo et al. 2019; Tong et al. 2019).

3D reconstructions from serial WSI are challenged by structural artifacts introduced during tissue preparation and imaging. Global 3D deformations arise from tissue extraction and processing, including shrinkage due to dehydration and fixation or distortions from mechanical stress and embedding (Al‐Kinani 2024). Sectioning and mounting introduce slice‐specific 2D deformations such as tearing, folding, shearing, and local warping (Al‐Kinani 2024). These morphological alterations are compounded by illumination variability (e.g., vignetting) and misalignment between tiles or serial sections, all of which hinder accurate spatial correspondence (Al‐Kinani 2024). Accurate quantitative analysis of whole slide images is critical for applications including feature extraction, tissue classification and automated cellular quantification (Hanna et al. 2020).

Raw microscopy data exhibits spatial pixel intensity inhomogeneities that can lead to stitching errors, intensity discontinuities, and compromise downstream analysis. To address these artifacts, several image correction methods have been developed, including corrected intensity distributions using regularized energy minimization (CIDRE) (Smith et al. 2015), BaSiC (Background and Shading Correction of Optical Microscopy Images) (Peng et al. 2017), and Vignetted Stitched‐Tile Adjustment using Morphological Adaptive Processing (VISTAmap) (Fung et al. 2025). However, these approaches can present reduced accuracy when few tiles are available or where tile‐specific correction may be required (Coster et al. 2014; Likar et al. 2000; Singh et al. 2014; Smith et al. 2015).

For stitching WSI tiles to create a single image, tools such as the ImageStitching Plugin in Fiji (Preibisch et al. 2009) and, more recently, ASHLAR (Alignment by Simultaneous Harmonization of Layer/Adjacency Registration) (Muhlich et al. 2022) have been developed, with ASHLAR specifically addressing residual misalignment from conventional tile‐stitching approaches (Chalfoun et al. 2017) (Zukić et al. 2021).

Histological 3D reconstruction methods have progressed from a simple sequential alignment to a reference section (Kiessling and Pichler 2010), which was prone to cumulative distortions such as the *z‐shift effect* (Yushkevich, Avants, et al. 2006). Without a global anatomical reference, some brain reconstruction frameworks use a pairwise‐only approach with iterative refinement (Woodward et al. 2018). More advanced frameworks integrate WSI with blockface photographs and 3D anatomical references from modalities such as magnetic resonance imaging (MRI), using affine and nonlinear transformations to achieve anatomically faithful reconstructions (Adler et al. 2014; Amunts et al. 2013; Chakravarty et al. 2006; Choe et al. 2011; Dauguet et al. 2009, 2007). Multimodal strategies such as the Possum Framework (Majka and Wojcik 2016) integrate multiple modalities into a unified spatial system and explicitly identify tissue tears and severe displacements as preprocessing challenges that must be addressed before reconstruction.

Deformable registration of serial histological sections is an ill‐posed problem that can be constrained by incorporating information about tissue boundaries to guide the registration. The level‐set method (LSM) is a numerical technique that embeds surface information into a signed distance function, representing surfaces implicitly as level‐sets to capture complex geometric and topological changes. This method has been previously used to embed surface‐based structural information in rigid body reconstruction of a rat brain (Kozinska et al. 1997) and to improve cortical surface alignment in human MRI brain images (Tardif et al. 2015). Motivated by the instability of unconstrained deformable registration in regions with weak anatomical contrast, we leverage LSM to introduce anatomically informed surface constraints that stabilize and guide the deformable alignment of serial histological sections.

This study presents an enhanced framework for 3D histological image reconstruction that addresses both tile‐level intensity inconsistencies and section‐level geometric distortions. We first introduce an overlap‐aware illumination correction for WSI that enables tile‐specific refinement while preserving global consistency. We then integrate LSM‐based anatomical constraints into the nonlinear registration stage of the Possum framework, stabilizing deformable alignment and mitigating common histological artifacts. Together, these advances aim to enhance reconstruction robustness and achieve more faithful restoration of native tissue morphology in 3D histological image reconstruction.

## 2 Materials

The animal tissue used for this study was obtained in accordance with the guidelines of the Canadian Council for Animal Care and approved by the McGill University Animal Care Committee.

### 2.1 Marmoset Imaging

This study utilized in vivo MRI and histological data from a single adult female common marmoset ( *Callithrix jacchus* (Linnaeus, 1758)) brain. Scanning was performed on a 3T‐PrismaFit scanner (Siemens, Germany) with a 16‐channel marmoset RF coil (Hori et al. 2018) (Takashima Seisakusho Co./Rogue Research Inc.). A *T*_1_‐weighted anatomical MRI scan was acquired using a magnetization prepared rapidly acquired gradient echo (MPRAGE) sequence with the following parameters: flip angle = 8°, TR = 2200 ms, TI = 706 ms, isotropic resolution of 0.3 × 0.3 × 0.3 mm^3^, two averages, with a total scan time of 13:31 min per acquisition. This was acquired at five different scan sessions over a three‐month period and averaged together. After the final MRI session, the brain was perfusion‐fixed, extracted, and placed in 4% paraformaldehyde, then cryosectioned in the coronal plane at 20 μm thickness. To match the resolution of the highest‐resolution MRI scan, 1 in every 15 sections was retained, resulting in 300 μm spacing between sections. During sectioning, blockface images were captured to facilitate accurate reconstruction and alignment. Sections were co‐labeled using a Hoechst 33,342 dye and Myelin Basic Protein, Tubulin Beta Class III, and Microtubule‐Associated Protein 2 antibodies (immunolabeling protocol detailed in Chitsaz et al. (2025)). WSI was performed using an ImageXpress High‐Content Imaging System with a 0.4 NA 10 × objective (Molecular Devices). Owing to variability in tissue section size, each histological section comprised between 2 and 256 image tiles, each acquired at a resolution of 0.67 μm/pixel and image size ranging from 11194 × 9380 to 18521 × 27673 pixels.

### 2.2 Synthetic Data: Banana

A *T*_2_‐weighted MR image of a curved banana with an isotropic voxel resolution of 1 mm^3^ was obtained from The Possum Framework (Majka and Wojcik 2016). This image was reconstructed to provide a synthetic, structured test object for visualizing alignment accuracy and identifying spatial distortions. The Possum Framework provides both a reference shape and a corresponding distorted version of the banana dataset (https://github.com/pmajka/poSSum/tree/master). To generate a distorted banana dataset with known transformation files, we performed a slice‐wise 2D rigid and affine registration between the distorted (fixed) and reference (moving) volumes. The resulting transformations, capturing translations, rotations, scaling, and shearing, created synthetically distorted sections. These were restacked into a 3D moving volume for reconstruction.

### 2.3 Mouse Data

To assess whether the proposed reconstruction framework generalizes to histological data that lacks a same‐specimen 3D reference volume, 10 parvalbumin‐stained histological sections from the Developmental Mouse Brain Atlas (DeMBA) (Bjerke et al. 2023) were reconstructed. A publicly available ex vivo *T*_2_*‐weighted MRI dataset of a C57BL/6J mouse brain (Ma et al. 2005, 2008) was used as the anatomical reference volume. The MRI acquisition protocol is detailed in (Ma et al. 2005). Briefly, imaging was performed on a formalin‐fixed C57BL/6J mouse brain using a 3D gradient echo sequence on a 17.6T magnet with the following parameters: 47 μm resolution, TR = 150 ms, TE = 7.5 ms, flip angle = 25°. The histological and MRI datasets were obtained from the EBRAINS platform (https://search.kg.ebrains.eu/instances/0c2adfec‐6225‐400b‐b438‐c52ae7fbde2f) and NeuroImaging Tools & Resources Collaboratory (https://www.nitrc.org/projects/c57bl_mr_atlas/), respectively.

## 3 Methods

### 3.1 Marmoset Whole Side Histology Vignette Correction and Tile Stitching

Tiles from all sections were first processed using BaSiC to estimate a specific flat‐field correction, which served as an initial baseline for subsequent section‐ and tile‐specific refinement. Full details of the tile‐level vignette correction are provided in Figure A1. Briefly, to improve local intensity consistency, overlapping regions between neighboring tiles were used to derive tile‐specific corrections. Processing was performed on a section‐by‐section basis, with the BaSiC flat‐field approximated by a fourth‐degree polynomial in both spatial dimensions for each tile. Pixel intensities within overlapping regions were then incorporated into a weighted least‐squares regression to refine the polynomial coefficients, enforcing agreement between overlapping tiles after correction. The BaSiC flat‐field estimate was weighted to 80% while the overlap consistency was weighted to 20%. To reduce computational burden, every 200th pixel was sampled for fitting, enabling execution on a desktop workstation with 64 GB of RAM. Algorithm performance was quantified using the Root Mean Squared Error (RMSE) computed between downsampled overlapping pixels in the log‐intensity domain across each section. Differences between methods were assessed using a Friedman chi‐squared test, followed by post hoc paired Wilcoxon tests with Holm correction.

Following intensity correction, tiles were stitched into a composite WSI using ASHLAR, producing reconstructed sections with pixels with a length of 0.67 μm.

### 3.2 Section Intensity Normalization

Intensity normalization of the serial sections was performed prior to reconstruction to reduce section‐to‐section intensity variations. Each histological slice was first denoised using a 2D 8 × 8 pixel median filter. Tissue‐specific masks for gray matter (GM) and white matter (WM) were manually segmented in ITK‐SNAP (Yushkevich, Piven, et al. 2006) and used to compute the median intensity for each tissue class. Slice‐wise correction maps were calculated by dividing each pixel by its corresponding tissue median intensity. To prevent overcorrection near tissue boundaries, the tissue masks were dilated, and peripheral regions were assigned a neutral correction factor. A smooth 2D polynomial surface was subsequently fitted to each correction map to enforce spatial smoothness. The effectiveness of intensity normalization was quantified using the coefficient of variation (CV) of voxel intensities within tissue regions across all slices.

### 3.3 Image Pre‐Processing

Prior to the image registration steps for the marmoset histological images, tile images in TIFF format (~1 GB each) underwent quality control. Quality control involved the removal of unusable slices and replacing them with neighboring sections when necessary. Full‐resolution images were downsampled by a factor of 100 and converted to PNG format to preserve the alpha channel. All image registrations were performed using Advanced Normalization Tools (ANTs) (Avants et al. 2008, 2011; Tustison et al. 2021). Landmark posts embedded in the blockface images facilitated registration. All posts were masked and aligned to a single masked reference post to ensure consistent imaging planes. After background removal, the transformation files from post‐registration were applied to the blockface images, converted to grayscale, and cropped to remove excess background.

### 3.4 Image Reconstruction Workflow

The reconstruction pipeline follows the established Possum framework (Majka and Wojcik 2016; Yushkevich, Piven, et al. 2006; Yushkevich, Avants, et al. 2006) and consists of a multi‐stage registration strategy designed to balance global anatomical fidelity with local alignment accuracy. First, each histological slice is affinely registered to its corresponding reference slice (pairwise registration). Second, sequential registration to neighboring slices refines local alignment. Third, a coarse‐to‐fine step combines the high‐frequency components of the sequential transformations with the pairwise alignment, preserving global brain geometry while maintaining local edge structure.

The marmoset *T*_1_‐weighted anatomical MRI was first rigidly and then affinely registered in 3D to the blockface volume to align the reference anatomy with the blockface cutting plane (overview in Figure A2). Blockface reconstruction proceeded using the coarse‐to‐fine registration strategy. Nonlinear registration was intentionally omitted to avoid introducing geometric distortions inherent to MRI, thereby preserving the anatomical fidelity of the blockface volume to which the labeled section images will better align. The reconstructed blockface volume subsequently served as the reference space for histological reconstruction.

### 3.5 Integration of LSM Into the Possum Framework

The Possum Framework concludes with a deformable registration stage to correct residual local distortions. To improve robustness and anatomical plausibility during deformable registration, LSM‐based constraints were incorporated directly into the deformable registration. Key anatomical structures, such as the full tissue (GM + WM) and WM regions (Figure 1B), were manually segmented in ITK‐SNAP for both moving and fixed images. The resulting masks for each region of interest (Figure 1C,D) were used to compute level‐set maps (Figure 1E,F) using SimpleITK (Lowekamp et al. 2013). In these maps, the surface boundary is defined by the zero‐level set, interior values are negative, and exterior values are positive.

**FIGURE 1:**
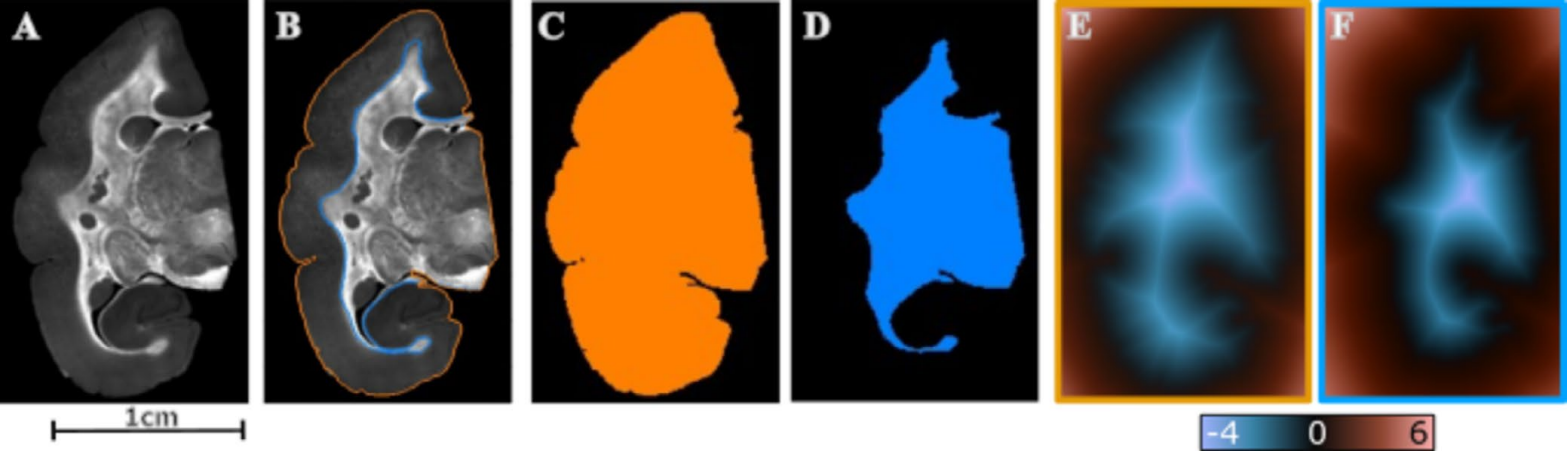
Generation of level‐set maps from histological sections. (A) Downsampled Hoechst 33342‐labeled section showing annotated key structures. (B) Manual white matter (WM) segmentation is outlined in blue, and full tissue (GM + WM) in orange. (C, D). Binary masks of the full tissue (GM + WM) and WM. (E, F) Corresponding level‐set representations. This workflow converts binary masks into level‐set maps for subsequent analysis.

These distance‐based representations encode both surface geometry and spatial context. The deformable registration step used a composite similarity metric combining mutual information (MI) between the moving and fixed image intensities and cross‐correlation (CC) between the corresponding level‐set representations. In contrast, the standard Possum framework relies solely on MI between fixed and moving image intensities. The inclusion of level‐set constraints provides explicit anatomical guidance, reducing instability in regions with weak tissue contrast.

The synthetic banana MRI, histological mouse and marmoset serial sections were processed using a modified Possum reconstruction pipeline to include LSM. Level‐set generation was adapted to each dataset. For the synthetic banana dataset, level‐set maps were computed from manually drawn segmentations of the full object and the internal structure (excluding the peel). For both the marmoset and mouse datasets, level‐set maps were computed from manually drawn segmentations of the full tissue (GM + WM) and WM regions.

Registration performance was evaluated using complementary similarity metrics. Normalized Mutual Information (NMI) was used to assess intermodal alignment by quantifying statistical dependence between image intensity distributions, with higher values indicating improved correspondence. Structural Similarity Index Measure (SSIM) quantified preservation of structural information based on local luminance, contrast, and spatial patterns, with values approaching 1 indicating stronger similarity. Both 2D and 3D variants of NMI and SSIM were computed, where 2D metrics represent slice‐wise averages and 3D metrics account for relative slice size. For intra‐modality comparisons, RMSE was calculated to quantify pixel‐wise intensity agreement, with lower values indicating improved alignment.

## 4 Results

### 4.1 Improved Tile Stitching and Vignette Correction Through Overlap‐Aware Methods

Overlap‐aware correction significantly reduced residual shading and tile gridding across histological sections. A Friedman test on RMSE in overlapping regions (112 sections each with four fluorescent labels provides 448 measurements) revealed a significant effect of correction method (*χ* ^2^(2) = 890.04, *p* < 2.2e‐16), with post hoc Wilcoxon tests showing differences between all pairs (*p* < 2.2e‐16). These improvements are illustrated in Figure 2, which highlights cases with few, intermediate, and many tiles.

**FIGURE 2:**
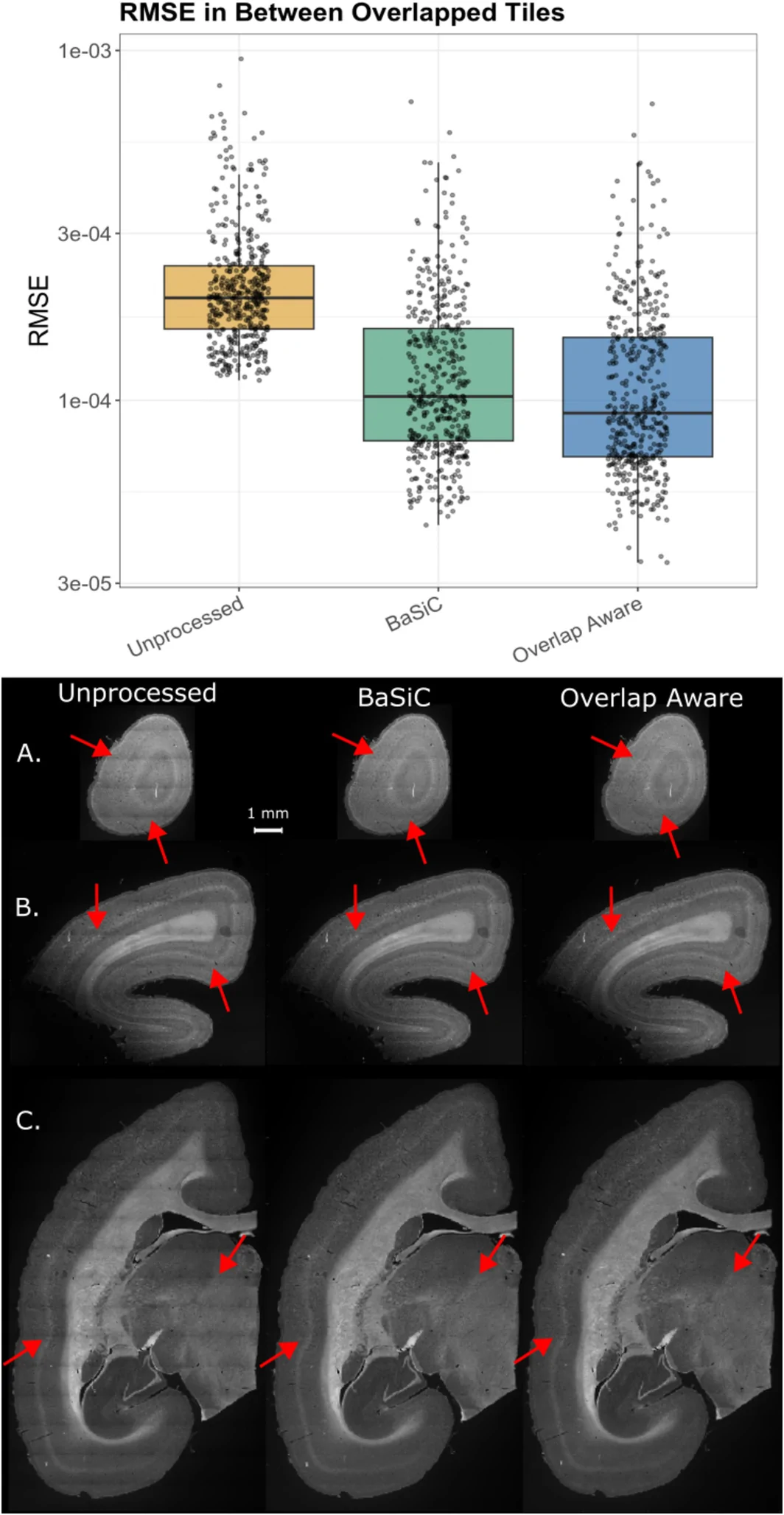
Tile stitching and vignette correction. Top: RMSE of log‐intensity differences across overlapping regions for 112 sections with four stains (448 measurements). Bottom: Representative sections showing correction performance. (A) Few tiles: BaSiC reduces shading but leaves gridding; overlap‐aware correction improves consistency. (B) Intermediate tiles: overlap‐aware method removes gridding artifacts. (C) Many tiles: overlap‐aware matches BaSiC in shading correction. Evaluation demonstrates improved intensity uniformity across multi‐tile sections. Red arrows highlight corresponding areas within the unprocessed, BaSiC, and overlap‐aware, demonstrating improved correction performance.

BaSiC substantially improved shading in the raw data across all sections, while the overlap‐aware correction further reduced residual tile gridding under sparse coverage. When few tiles were available, the overlap‐aware method mitigated apparent gridding artifacts, though some residual effects remained due to its weighting toward the BaSiC solution (Figure 2A). With an intermediate number of tiles, the overlap‐aware correction successfully removed tile gridding in cases where BaSiC alone was insufficient (Figure 2B). For larger image sections containing many overlapping tiles, the overlap‐aware method performed comparably to BaSiC, effectively correcting shading artifacts within imaging tiles (Figure 2C).

### 4.2 Homogenous Histological Sections Through Normalization

Application of the normalization procedure reduced intensity variability across slices (Figure 3). Specifically, the averaged CV within sections decreased from 0.392 in the unnormalized images to 0.246 after normalization, indicating a more homogeneous volume and effective removal of inter‐slice intensity differences. Residual variation is expected because tissue classes exhibit inherent intensity differences.

**FIGURE 3:**
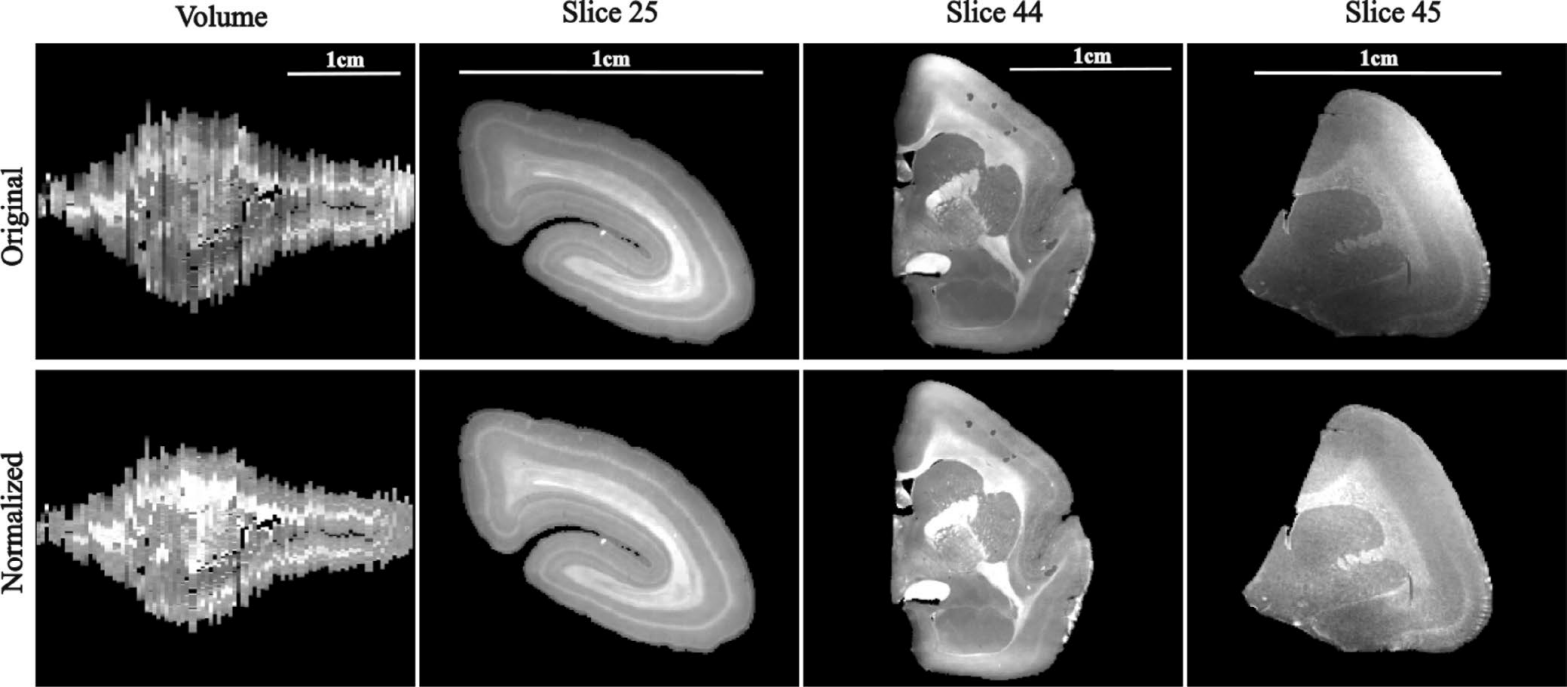
Comparison of original (top) and intensity‐normalized (bottom) marmoset histological sections. The 3D section stack (left) and representative coronal slices 25, 44, and 45 illustrate the reduction in inter‐ and intra‐section intensity variation following normalization, with improved tissue contrast consistency across slices.

### 4.3 Enhanced Alignment of Histology to Blockface Using LSM‐Constrained Registration

Incorporating LSM constraints into the deformable registration stage improved alignment of histological sections to blockface images. Qualitative assessment revealed that, relative to the coarse‐to‐fine baseline, the LSM‐integrated framework reduced local misalignment and better preserved the anatomical boundaries of GM and WM. Quantitatively, 2D/3D NMI increased from 0.4470/0.3929 to 0.4946/0.4301, and SSIM increased from 0.8022/0.7220 to 0.8297/0.7582, corresponding to improvements of approximately 10% in NMI and 3%–5% in SSIM. In contrast, the deformable method without LSM achieved more modest gains (NMI: ~3%; SSIM: ~0%–2%). These effects are illustrated in Figures 4 and 5 and summarized in Table 1.

**FIGURE 4:**
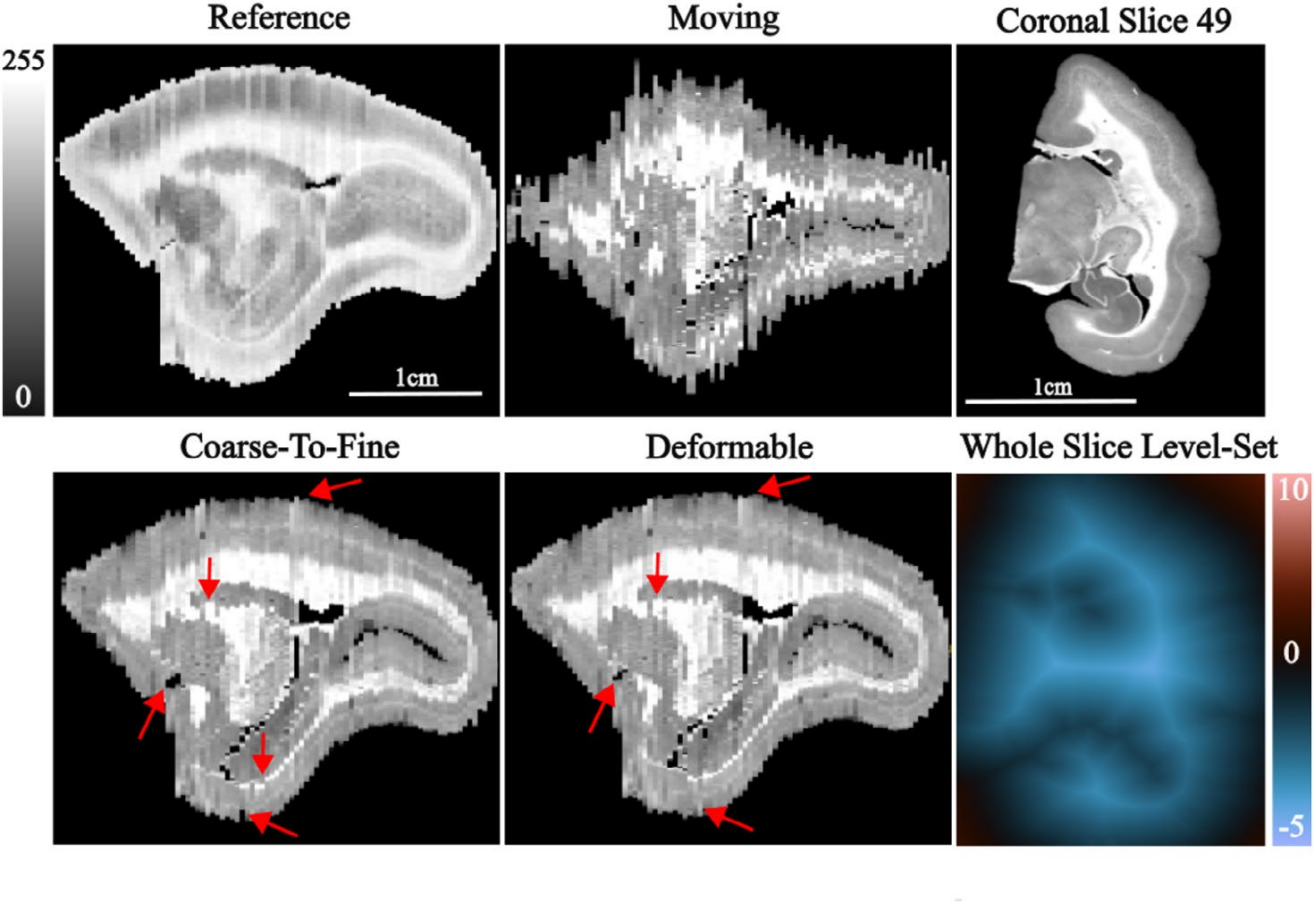
Results of the marmoset histology reconstruction pipeline across registration stages. Top row: reference blockface volume, moving stack of fluorescently labeled slices (unregistered), and coronal slice 49 of the coarse‐to‐fine volume. Red arrows demonstrate areas of remaining structural inconsistency. Bottom row: coarse‐to‐fine and LSM deformable registration results, with the corresponding full tissue (GM + WM) level‐set. Red arrows demonstrate improved structural consistency from LSM deformable registration.

**FIGURE 5:**
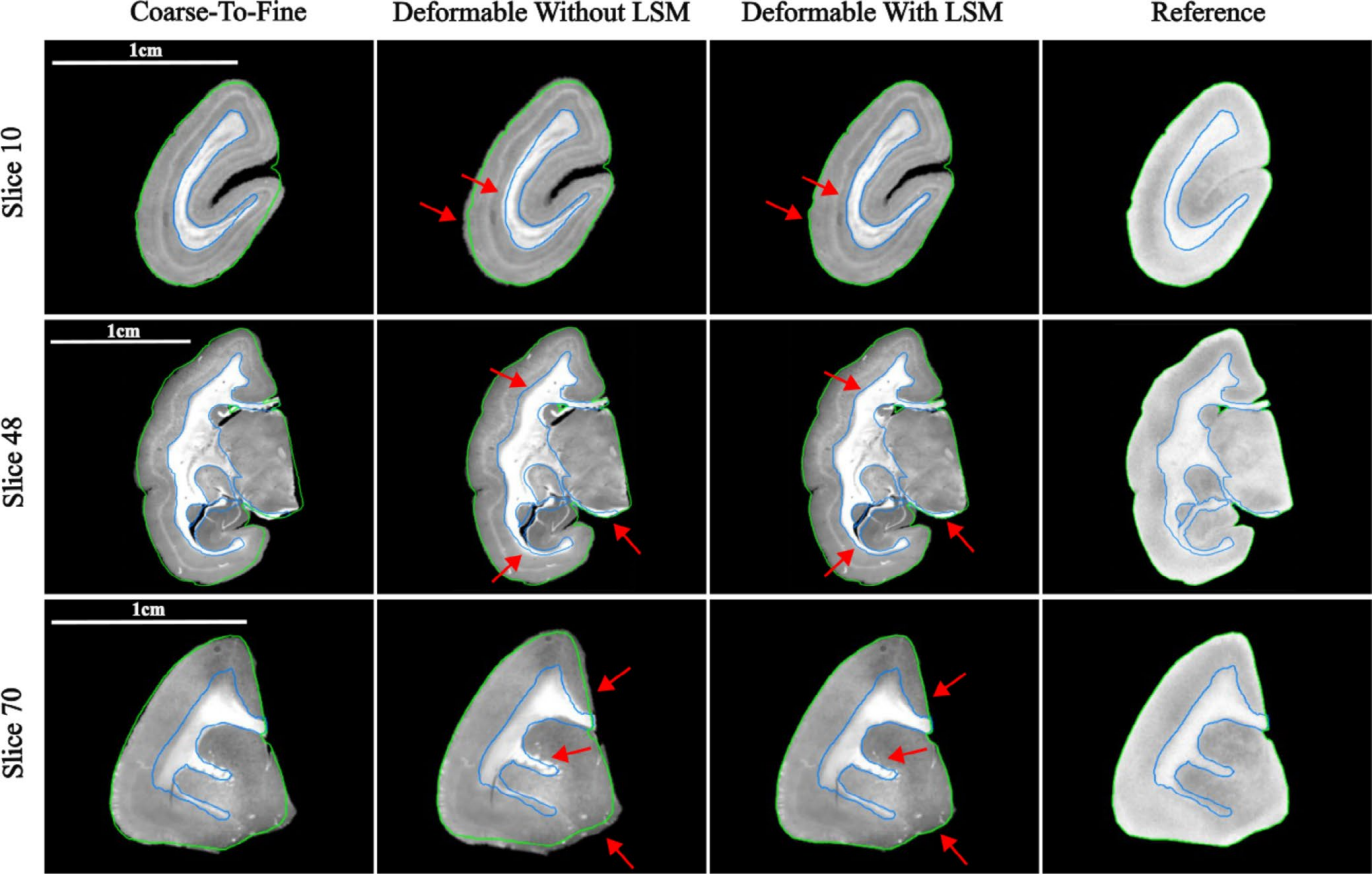
Comparison of registered marmoset labeled sections across registration stages for representative coronal slices 10, 48, and 70. Columns show coarse‐to‐fine, deformable without LSM, deformable with LSM, and the reference blockface photography slice. Green and blue contours indicate the GM + WM and WM boundaries of the reference, respectively. Red arrows highlight regions of residual misregistration in the deformable without LSM column, while the same regions in the deformable with LSM column demonstrate improved alignment.

**TABLE 1:** 2D and 3D NMI and SSIM scores at each stage of image registration for the marmoset histological reconstruction pipeline.

Stage	NMI 2D avg.	3D NMI	SSIM 2D avg.	3D SSIM
Pairwise	0.4504	0.3935	0.8013	0.7245
Sequential	0.1872	0.2056	0.6988	0.5834
Coarse‐to‐Fine	0.4470	0.3929	0.8022	0.7220
Deformable without LSM	0.4617	0.4048	0.8030	0.7333
Deformable with LSM	0.4946	0.4301	0.8297	0.7582

Slice‐wise NMI distributions, summarized in Figure 6, were compared across the coarse‐to‐fine, LSM‐integrated deformable stage, and the deformable method without LSM. A Friedman test indicated a significant effect of reconstruction strategy on NMI (*χ* ^*2*^(2) = 155.38, *p* < 2.2 × 10^−16^). Post hoc pairwise comparisons using the Wilcoxon signed‐rank test with Bonferroni correction showed that deformable with LSM significantly outperformed both coarse‐to‐fine (*p* < 2 × 10^−16^) and the deformable method without LSM (*p* < 2 × 10^−16^), while the deformable method without LSM also performed significantly better than coarse‐to‐fine (*p* = 5.3 × 10^−9^).

**FIGURE 6:**
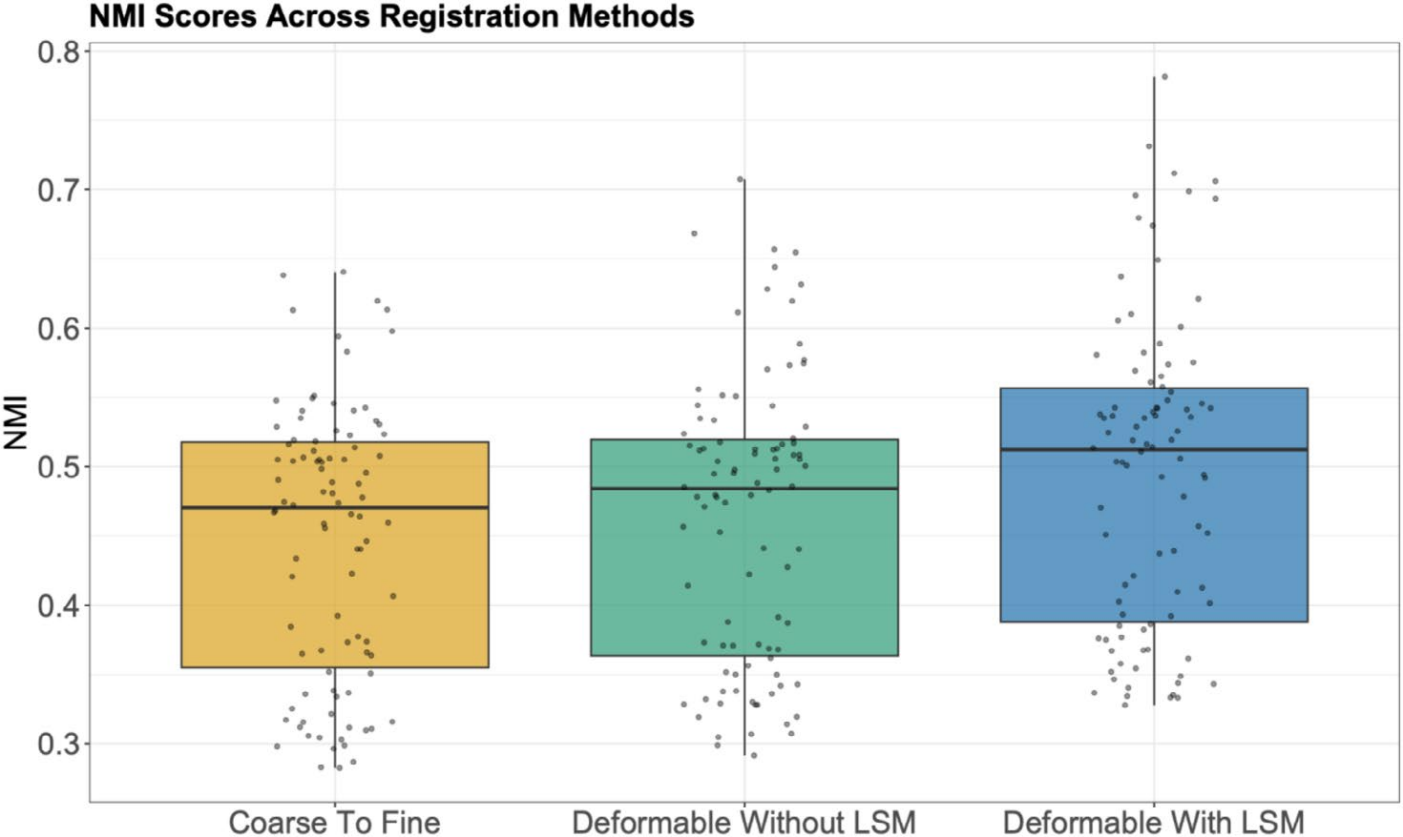
Boxplot of 2D NMI scores across coarse‐to‐fine, deformable without LSM, and deformable with LSM registration stages during the histological marmoset image reconstruction.

### 4.4 LSM‐Integrated Reconstruction in Banana Dataset

Incorporating LSM into the deformable registration stage improved anatomical continuity and structural fidelity in the reconstructed banana MRI volume. Compared with the standard Possum framework (without LSM), the LSM‐integrated approach reduced local misalignment and better preserved the internal structure of the synthetic object. These improvements are illustrated in Figure 7. Quantitative evaluation is summarized in Table A1. The LSM‐integrated framework achieved an RMSE of 1.676%, compared with 1.971% without LSM. Relative to the coarse‐to‐fine reconstruction baseline (2.286%), this corresponds to a 26.7% improvement. When compared directly with the deformable without LSM, the LSM‐integrated method shows a 15.0% improvement in RMSE. These improvements are further validated through absolute error maps (Figure 7H) between deformable with LSM (Figure 7F) and deformable without LSM (Figure 7G). These maps demonstrate reduced intensity differences when LSM constraints are applied, particularly in regions of high curvature.

**FIGURE 7:**
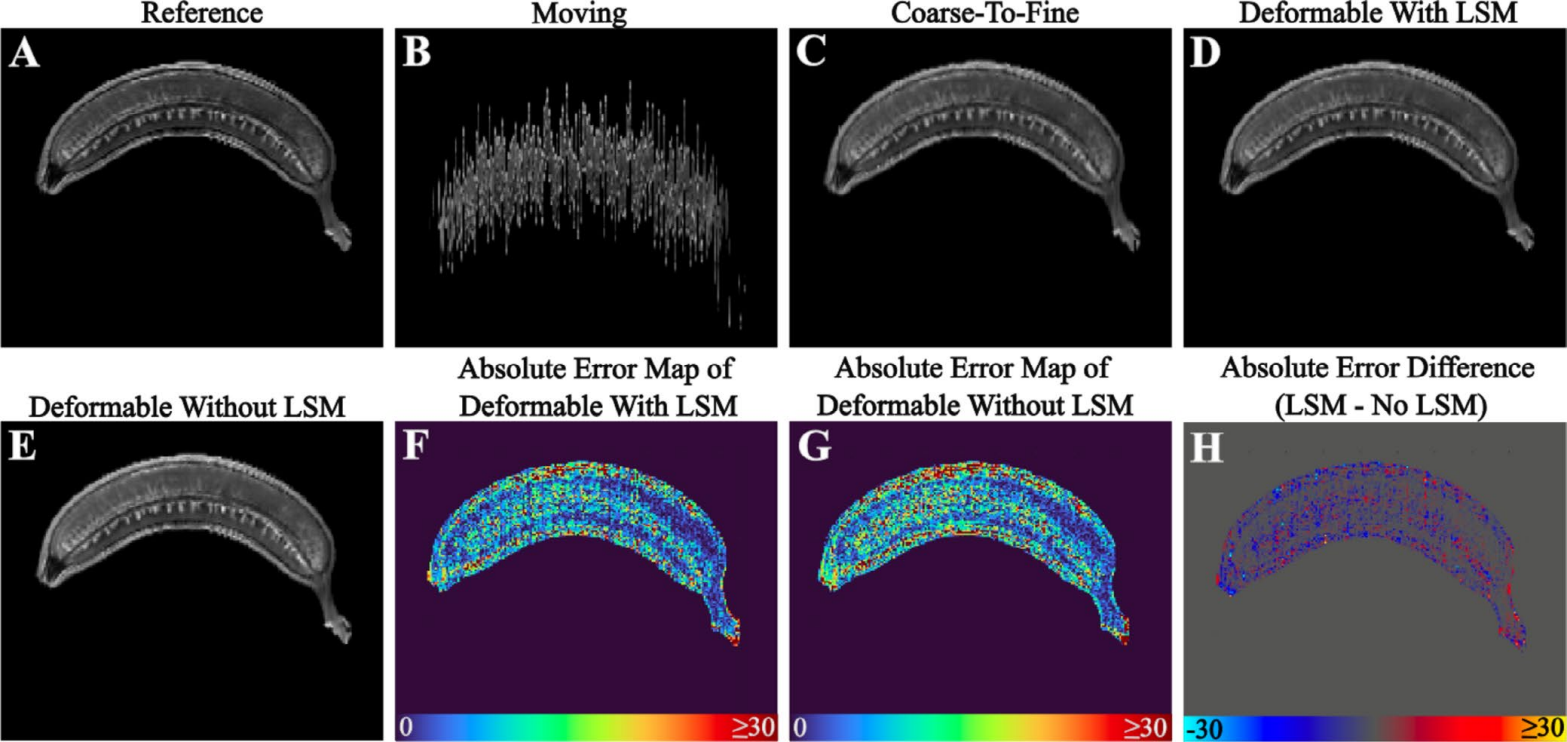
Reconstruction of the synthetic MRI banana phantom: (A) reference volume; (B) unregistered moving volume; (C) coarse‐to‐fine registration result; (D) deformable with LSM registration result; (E) deformable registration without LSM; (F) absolute error map of deformable with LSM; (G) absolute error map of deformable without LSM; (H) absolute error difference between deformable with LSM and deformable without LSM.

### 4.5 LSM‐Integrated Framework Enhances Cross‐Specimen 3D Histological Reconstruction

Using unmatched MRI and histology from different mouse brains, the incorporation of LSM into the deformable registration stage substantially improved cross‐specimen 3D histological alignment, stabilizing the deformation and improving correspondence of pial boundaries and WM regions. Relative to the coarse‐to‐fine baseline, the LSM‐integrated framework increased 2D/3D NMI from 0.3010/0.2821 to 0.3462/0.3238 and SSIM from 0.4993/0.4914 to 0.5105/0.5007, corresponding to gains of ~14% in NMI and ~2% in SSIM. In comparison, the deformable method without level‐sets produced more modest improvements (NMI: 0.3042/0.2852; SSIM: 0.4999/0.4923). These effects are illustrated in Figure 8 and summarized in Table A2, highlighting the robust enhancement in volumetric and structural alignment achieved with LSM guidance.

**FIGURE 8:**
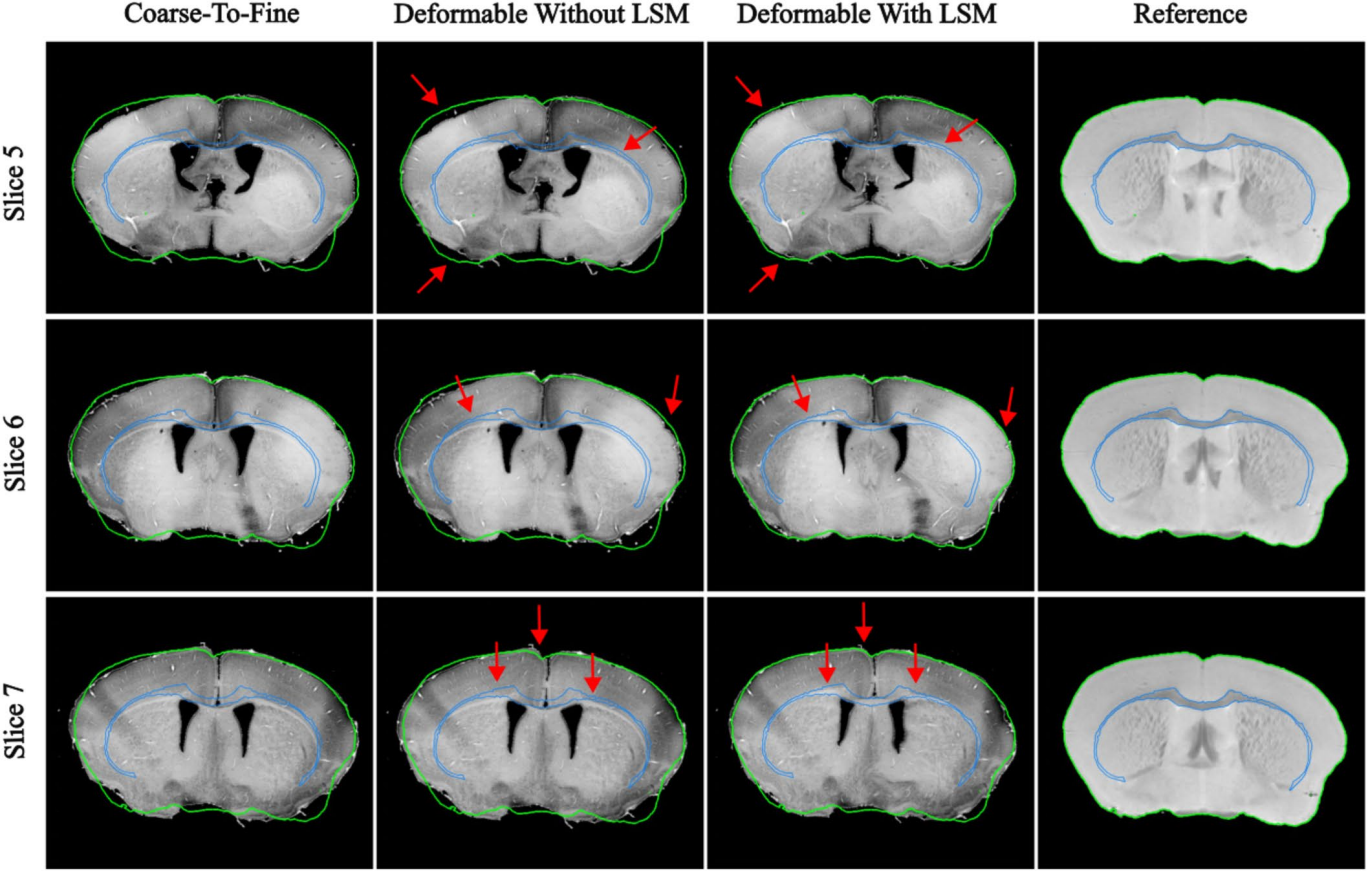
Results of the registration workflow applied to parvalbumin‐stained mouse histology, using a *T*_2_*‐weighted MRI volume from a different subject as reference, for representative coronal slices 5, 6, and 7. Columns show coarse‐to‐fine, deformable without LSM, deformable with LSM, and the reference slice. Green and blue contours indicate the GM + WM and WM boundaries of the reference, respectively. Red arrows highlight regions where deformable registration is expected to improve alignment. In the deformable without LSM column, these regions show residual misregistration, while the deformable with LSM column demonstrates improved alignment at the same locations.

## 5 Discussion

In this work, we presented an integrated workflow combining vignette tile correction, slice normalization, and level‐set–guided deformable registration to improve the 3D reconstruction of serial histological sections. Together, these components address common sources of error in histological reconstruction, including spatial intensity inhomogeneity within tiles, variability between sections, and instability in deformable registration. Across multiple validation experiments, including synthetic datasets, blockface registration, and cross‐specimen reconstruction, the proposed framework improved anatomical continuity and structural correspondence, yielding consistent gains in RMSE, NMI, and SSIM.

Microscopy data frequently exhibit spatial intensity inhomogeneities that can introduce stitching artifacts, intensity discontinuities, and errors in downstream analysis (Leong et al. 2003). Existing approaches, including low‐rank shading models and methods such as BaSiC (Peng et al. 2017), provide effective correction when sufficient tile overlap is available but may be limited under sparse tile coverage or when tile‐specific corrections are required (Smith et al. 2015). Our results demonstrate that overlap‐aware vignette correction effectively reduces residual gridding artifacts across varying tile counts. When many tiles were present, the method performed comparably to BaSiC, while under sparse coverage, it provided improved correction by explicitly leveraging overlap information between neighboring tiles. These findings suggest that incorporating overlap information provides a robust and adaptable approach for shading correction in whole‐slide imaging, improving both shading uniformity and tile alignment across diverse acquisition settings.

Inter‐section intensity variability is another major challenge in serial histology reconstruction. Differences in section thickness, stain penetration, fixation quality, and slide preparation can produce large variations in overall intensity between slices (Al‐Kinani 2024). Without appropriate normalization, these variations can bias similarity metrics used during registration, causing algorithms to interpret intensity differences as structural discrepancies and ultimately degrading alignment accuracy (Maes et al. 1997). Conventional global normalization approaches standardize overall intensity distributions but may compress biologically meaningful contrast between tissue compartments. In contrast, the proposed approach normalizes GM and WM independently using their respective median intensities, preserving intrinsic tissue contrast while reducing inter‐section variability. Modeling the correction factor with a low‐order polynomial further enforces spatial smoothness, allowing macroscopic intensity variation to be corrected while maintaining fine anatomical detail.

To address geometric distortions that arise during sectioning and mounting, we introduced a deformable registration framework incorporating LSM as structural constraints. Across all datasets, integrating LSM into the deformable stage consistently improved performance relative to both the coarse‐to‐fine baseline and deformable approaches without LSM guidance. In the marmoset histology‐to‐blockface experiment, LSM integration increased both 2D and 3D similarity metrics and visually stabilized deformations in regions prone to local misalignment. In the synthetic banana dataset, inclusion of LSM reduced RMSE, indicating improved structural continuity and reduced local misalignment. Similarly, in the mouse histology reconstruction, LSM‐guided registration improved NMI and SSIM, reflecting better correspondence of pial and WM boundaries. These results suggest that augmenting deformable registration with structural constraints derived from level sets provides a consistent and meaningful improvement in alignment accuracy.

An advantage of this framework is its flexibility in incorporating reference information. While optimal results are expected when the reference image originates from the same specimen, such as blockface imaging or MRI, the method can also operate using references from different subjects or modalities, including computed tomography (CT). In this study, level‐set maps were generated from manually segmented masks, but this step could also be implemented using automated segmentation tools or existing anatomical atlases. Consequently, the approach is not limited to histological brain reconstruction but may apply to other serial imaging datasets where distortions must be corrected.

Three‐dimensional reconstruction from serial histological sections is an increasingly active area of research, with large‐scale initiatives such as BigBrain (Amunts et al. 2013) and BigMac (Howard et al. 2022), and maps of cyto‐ and fiber architecture of the human brain (Alkemade et al. 2022). This work also spans region‐specific reconstructions of structures including the hippocampus (Adler et al. 2014), basal ganglia and thalamus (Chakravarty et al. 2006), and non‐human primate brains such as the owl monkey (Choe et al. 2011) and baboon (Dauguet et al. 2007). Other studies have applied similar reconstruction approaches to non‐neural tissues, including prostate (Gibson et al. 2013) and liver (Song et al. 2013). While these efforts demonstrate the feasibility of high‐resolution 3D reconstruction, many rely on dataset‐specific processing pipelines, extensive manual intervention, or carefully controlled acquisition protocols. As a result, generalizability across tissues, staining protocols, and experimental conditions remains a persistent challenge. By incorporating level‐set constraints within the deformable stage, the framework presented here provides a structurally guided mechanism to stabilize reconstruction without relying on extensive manual landmark placement or dataset‐specific parameter tuning. Importantly, the ability to incorporate level‐set information within defined regions of interest allows the method to extend beyond whole‐brain reconstruction to organ‐specific applications.

### 5.1 Limitations

Several limitations of the proposed framework warrant consideration. The overlap‐aware intensity correction relies on the presence of sufficient and accurately registered overlap regions between tiles. When overlap is minimal, tiling geometry is irregular, or tile positions are inaccurately estimated, the correction may become under‐constrained or propagate boundary inconsistencies across tiles. In addition, the method assumes that illumination inhomogeneity varies smoothly across the field of view. While appropriate for vignetting and large‐scale shading effects, this assumption may limit the correction of abrupt illumination changes or tile‐specific acquisition artifacts.

The tissue‐class‐specific intensity normalization reduces inter‐slice variability but currently relies on manually generated segmentations, introducing additional labor and potential observer‐dependent variability. Although automated segmentation methods could mitigate this limitation, they were not evaluated in this study. Furthermore, the normalization assumes relatively consistent intra‐class intensity distributions across sections. While this stabilizes similarity metrics and improves alignment robustness, it may attenuate subtle biologically meaningful contrast variations in regions with heterogeneous staining or pathology.

Finally, although LSM provides a strong structural constraint during deformable registration, it cannot fully compensate for distortions inherent to histological processing. When external reference atlases are used, anatomical differences between specimens may introduce bias, particularly in regions with substantial inter‐subject variability. Optimal performance is therefore expected when specimen‐specific references, such as blockface imaging or same‐subject MRI, are available. Acquiring a post‐mortem MRI prior to tissue processing may further reduce registration bias by providing a subject‐specific reference that captures fixation‐ and shrinkage‐related distortions before histological sectioning. Additionally, deformable registration cannot reconstruct missing tissue or fully correct severe artifacts such as tears, folds, or compression introduced during sectioning.

In this study, we presented an integrated framework for 3D histological reconstruction that combines overlap‐aware tile correction, tissue‐specific intensity normalization, and LSM‐guided deformable registration. Across synthetic, single‐subject, and cross‐specimen datasets, the proposed workflow improved structural fidelity, volumetric alignment, and inter‐slice intensity consistency relative to conventional approaches. By providing a robust and flexible reconstruction pipeline, this framework enables reproducible 3D reconstruction of serial histological data and supports downstream quantitative analysis across tissues, modalities, and species.

## Author Contributions

**Emily L. De Leon Alvarado:** conceptualization, investigation, writing – original draft, methodology, validation, visualization, formal analysis, data curation. **Ilana R. Leppert:** writing – review and editing, data curation. **Christine L. Tardif:** writing – review and editing, funding acquisition, supervision. **G. Bruce Pike:** writing – review and editing, supervision. **Yangyingqi Zhou:** methodology, writing – review and editing. **Jennifer S. W. Campbell:** writing – review and editing. **Daryan Chitsaz:** writing – review and editing, data curation. **Timothy E. Kennedy:** writing – review and editing, supervision. **Christopher D. Rowley:** investigation, funding acquisition, supervision, project administration, writing – review and editing, data curation, formal analysis.

## Funding

This work was supported by the Natural Sciences and Engineering Research Council of Canada (RGPIN‐2024‐06106).

## Conflicts of Interest

The authors declare no conflicts of interest.

## Figures and Tables

**FIGURE A1:**
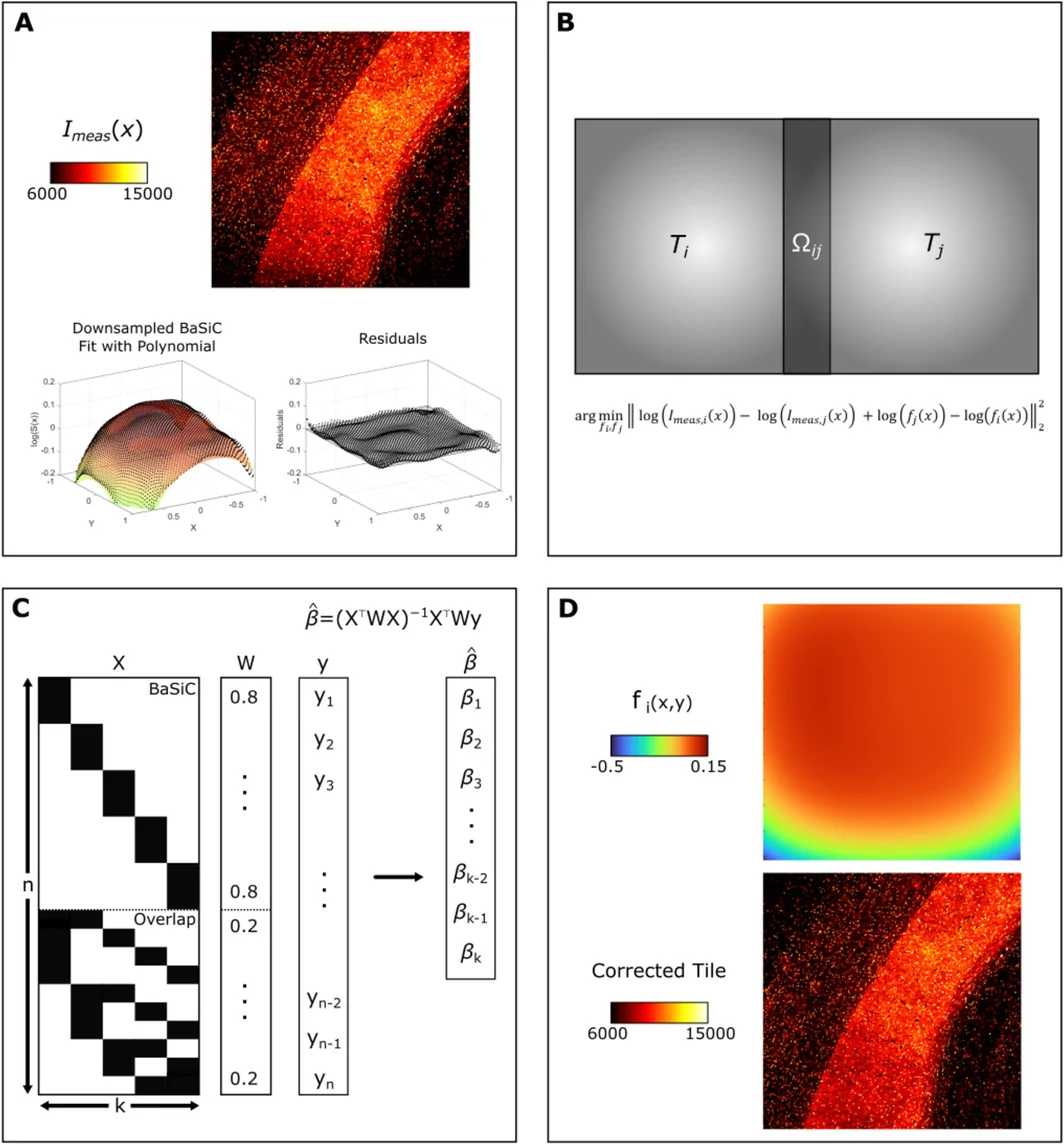
A visual representation of the intensity correction algorithm. (A) The spatial shading profile is evident in a representative image tile. The log‐transformed shading field estimated by BaSiC is shown, with every 200th pixel retained as a scatter point for visualization. The residual plot demonstrates that a fourth‐degree polynomial captures the majority of the spatial variation. (B) In whole slide imaging, adjacent tiles overlap spatially; intensity differences within the overlap region $\Omega_{\mathrm{ij}}$ provide constraints enforcing consistency between neighboring tiles. (C) Global illumination estimates derived from BaSiC and local overlap‐consistency constraints are combined into a single weighted linear regression problem. The design matrix X incorporates the BaSiC estimate in a block‐structured form together with overlap‐consistency terms, while the weighting matrix W controls the relative contribution of each constraint. The response vector y contains either BaSiC pixel values or intensity differences computed from overlapping tile pairs. (D) The resulting tile‐specific correction maps are applied in the log domain to produce corrected tiles.

**FIGURE A2:**
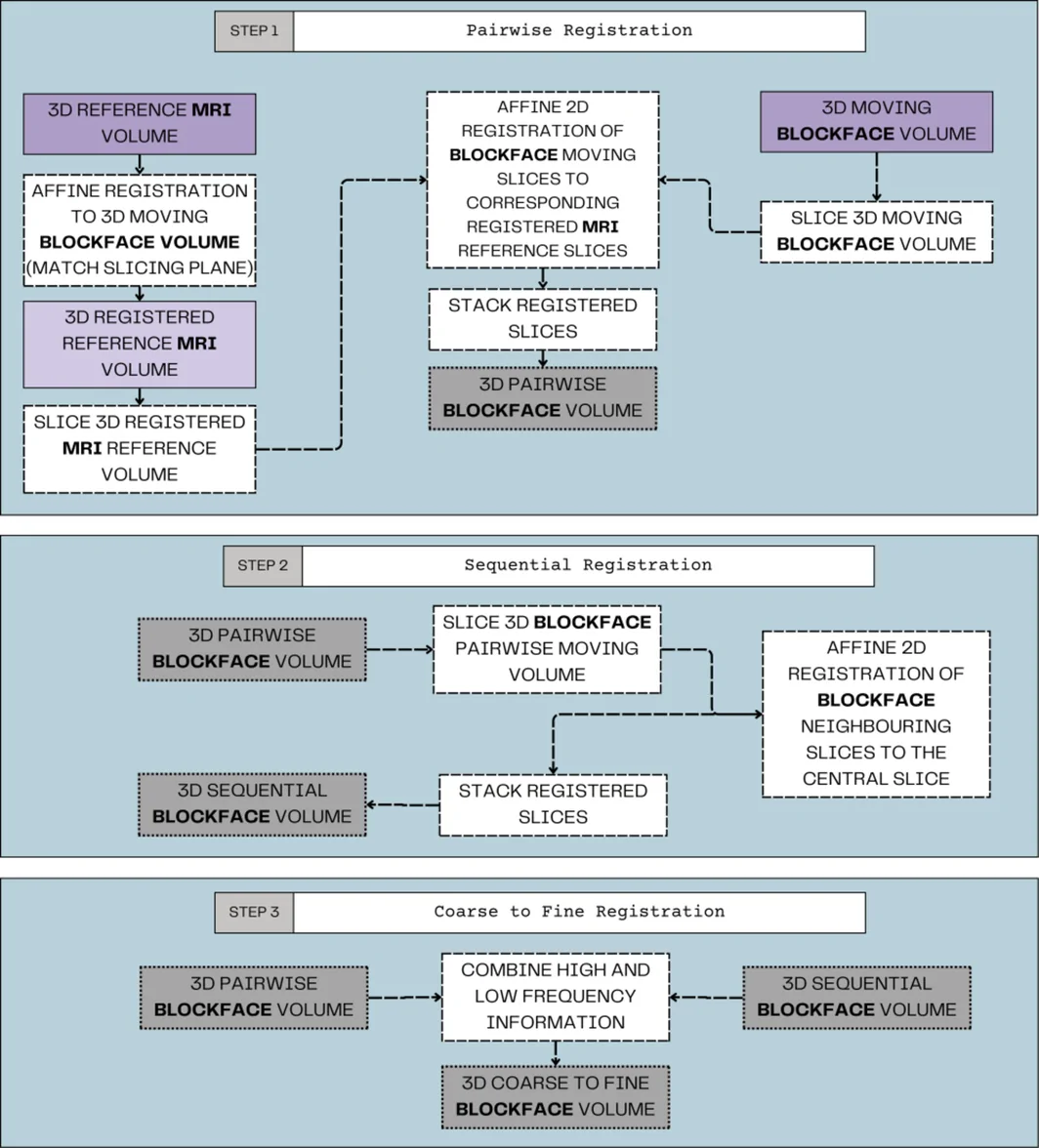
Workflow of blockface and MRI registration for marmoset histology. Purple boxes denote input volumes, white dashed boxes denote processing steps, and dark gray boxes denote output volumes. *Step 1: Pairwise Registration*: The 3D *T*_1_‐weighted MRI reference volume is affinely registered to the 3D blockface volume, followed by slice‐wise affine registration of blockface slices to the corresponding registered MRI slices, producing a 3D pairwise blockface volume. *Step 2: Sequential Registration*: Neighboring blockface slices are sequentially registered to a central slice and stacked to create a 3D sequential blockface volume. *Step 3: Coarse to Fine Registration*: High and low frequency information from the pairwise and sequential volumes are combined to generate the final 3D blockface volume with improved anatomical continuity.

## Data Availability

The code supporting the methods in this study is available for download from the GitHub repository: https://github.com/ResonanceImagingLab/Histology3DRecon. The data that support the findings of this study are available from the corresponding author upon reasonable request.

## Appendix A

### Vignette Correction Theory

The observed intensity $\left(I_{\text{meas}}\right)$ at spatial location *x* in a microscopy image can be modeled as follows:

$$I_{\text{meas}}\left(x\right)=I_{\text{true}}\left(x\right)\cdot S\left(x\right)+D\left(x\right)$$

where $I_{\text{true}}\left(x\right)$ is the true signal, *S*(*x*) is the spatially varying flat‐field (illumination), and *D*(*x*) is the dark‐field offset (e.g., thermal noise or stray light). The BaSiC algorithm estimates *S*(*x*) and *D*(*x*) using a low‐rank and sparse decomposition model.

Assuming a sufficiently high signal‐to‐noise ratio, the darkfield component can be neglected. Under this assumption, the model simplifies to a purely multiplicative form, which can be converted to an additive model via a logarithmic transformation:

$$\log\left(I_{\text{meas}}\left(x\right)\right)=\log\left(I_{\text{true}}\left(x\right)\right)+\log\left(S\left(x\right)\right)$$

In the log domain, estimation of the shading field *S*(*x*) can be posed as a least‐squares minimization problem:

$$\hat{S}=\arg\min_{S}{\left\|\log\left(I_{\text{meas}}\left(x\right)\right)-\left[\;\log\left(I_{\text{true}}\left(x\right)\right)+\log\left(S\left(x\right)\right)\right]\;\right\|}_{2}^{2}$$

This objective is minimized when the estimated shading field, applied multiplicatively to the unknown true image, reproduces the measured image. Because both *S*(*x*) and *I*_*true*_(*x*) are unknown, this problem is ill‐posed without additional constraints. Here, BaSiC provides an initial estimate of *S*(*x*).

*Overlap‐Aware Intensity Correction*: In WSI, neighboring tiles are typically acquired with an overlapping region. These overlapping regions provide additional constraints that can be exploited to refine illumination correction on a per‐tile basis. Rather than relying solely on a global shading field *S*(*x*), we introduce tile‐specific correction functions $f_{i}\left(x\right)$ for tile *T*_*i*_.

Let *T*_*i*_ and *T*_*j*_ be two adjacent image tiles with an overlapping region $\Omega_{\mathrm{ij}}\subset T_{i}\cap T_{j}.$ After BaSiC correction, residual intensity differences may persist in $\Omega_{\mathrm{ij}}$, that is, $I_{\text{corr}}^{\left(i\right)}\left(x\right)\neq I_{\text{corr}}^{\left(j\right)}\left(x\right)$ for $x\subset \Omega_{\mathrm{ij}}$.

We seek tile‐specific correction functions that minimize these discrepancies. In the log domain, this can be formulated as follows:

$$\arg\min_{f_{i},f_{j}}{\left\|\left[\;\log\left(I_{\text{meas},i}\left(x\right)\right)-\log\left(f_{i}\left(x\right)\right)\right]-\left[\;\log\left(I_{\text{meas},j}\left(x\right)\right)-\log\left(f_{j}\left(x\right)\right)\right]\;\right\|}_{2}^{2},x\subset \Omega_{\mathrm{ij}}$$

Rearranging terms yields the equivalent objective:

$$\arg\min_{f_{i},f_{j}}{\left\|\;\log\left(I_{\text{meas},i}\left(x\right)\right)-\log\left(I_{\text{meas},j}\left(x\right)\right)+\log\left(f_{j}\left(x\right)\right)-\log\left(f_{i}\left(x\right)\right)\right\|}_{2}^{2}$$

Since this only fits the overlapped regions, it only informs the fit about the perimeter of the tiles. Consequently, an initial global estimate (e.g., from BaSiC) remains necessary to constrain the correction in the central regions of each tile.

*Polynomial Parameterization of Tile‐Specific Corrections*: Each tile‐specific correction function $f_{i}\left(x,y\right)$ is approximated as a two‐dimensional polynomial of degree four in the spatial coordinates:

$$f_{i}\left(x,y\right)=\sum_{l=0}^{4}\sum_{m=0}^{4}a_{l,m}x^{l}y^{m}$$

This basis contains 15 coefficients per tile, yielding a parameter vector of length $15N_{t}$, where $N_{t}$ is the total number of tiles. Spatial coordinates are defined relative to the tile center, such that $\left(x,y)=(0,0\right)$ corresponds to the tile midpoint.

*Design Matrix Construction and Weighted Least Squares Estimation*: To estimate all tile‐specific coefficients jointly, we construct a single linear system encompassing both the global BaSiC constraint and the overlap consistency constraints.

First, a global shading field $S\left(x,y\right)$ is estimated using BaSiC across all tiles and sections for a given stain, restricted to regions containing tissue. For each pixel location, the polynomial basis functions are evaluated to populate the design matrix. If all pixels were included, the resulting matrix would have dimensions $N_{p}\times 15N_{t}$, where $N_{p}$ is the total number of pixels across all tiles. The matrix is block‐diagonal in structure, with nonzero entries for tile i occupying columns $15\left(i-1\right)+1$ through 15i. The corresponding response vector contains the values of $\log S\left(x,y\right)$, replicated across tiles.

To incorporate overlap constraints, additional rows are appended to the design matrix for pixels in overlapping regions $\Omega_{\mathrm{ij}}$. Polynomial basis terms corresponding to tile $T_{i}$ enter with a negative sign, while those for tile $T_{j}$ enter with a positive sign, reflecting the subtraction in the overlap objective. The response variable for these rows is given by:

$$\log\left(I_{\text{meas},i}\left(x\right)\right)-\log\left(I_{\text{meas},j}\left(x\right)\right)$$

To reduce memory usage, only every 200th pixel is retained from both the global and overlap conditions. Additionally, a Gaussian smoothing kernel with a standard deviation of 1.5 pixels is applied to the images prior to sampling, reducing the influence of high‐frequency noise on the overlap consistency constraint.

As the global BaSiC constraint contributes substantially more rows to the system than the overlap constraint, the resulting fit is naturally biased toward the global solution. To balance these contributions, we apply weighted least squares regression, assigning 80% of the total weight to the BaSiC‐derived global constraint and 20% to the overlap consistency constraint. All tile‐specific polynomial coefficients are then estimated simultaneously via weighted linear regression.

With tile‐specific coefficients determined, correction maps are generated by evaluating the polynomial $f_{i}\left(x,y\right)$ at each pixel location within tile i. These correction maps are subtracted from the log‐transformed measured images. Finally, the corrected images are exponentiated to return them to the original intensity domain. This takes approximately 3 min for a 10 × 11 tile image, with each tile possessing 2048 × 2048 pixels.

**TABLE A1:** RMSE (%) scores at each stage of image registration for the synthetic MRI banana phantom reconstruction.

Stage	RMSE (%)
Pairwise	2.286
Sequential	9.086
Coarse to Fine	2.286
Deformable without LSM	1.971
Deformable with LSM	1.676

**TABLE A2:** NMI and SSIM scores at each stage of image registration for the mouse histological reconstruction pipeline.

Stage	NMI 2D avg.	3D NMI	SSIM 2D avg.	3D SSIM
Pairwise	0.2974	0.2789	0.4959	0.4874
Sequential	0.2989	0.2800	0.4992	0.4911
Coarse‐to‐Fine	0.3010	0.2821	0.4993	0.4914
Deformable without LSM	0.3042	0.2852	0.4999	0.4923
Deformable with LSM	0.3462	0.3238	0.5105	0.5007
